# Combination of resolvin E1 and lipoxin A4 promotes the resolution of pulpitis by inhibiting NF‐κB activation through upregulating sirtuin 7 in dental pulp fibroblasts

**DOI:** 10.1111/cpr.13227

**Published:** 2022-04-11

**Authors:** Xiaochen Liu, Chunmeng Wang, Liping Pang, Liangliang Pan, Qi Zhang

**Affiliations:** ^1^ Department of Endodontics, School & Hospital of Stomatology Tongji University, Shanghai Engineering Research Center of Tooth Restoration and Regeneration Shanghai China

## Abstract

**Objectives:**

To determine whether the combination of resolvin E1 (RvE1) and lipoxin A4 (LXA4) could promote resolution of pulpitis and to investigate the mechanism.

**Materials and Methods:**

Preliminary screening was first conducted in four specialized pro‐resolving mediators (SPMs). Real‐time quantitative polymerase chain reaction, western blotting, enzyme‐linked immunosorbent assay and double‐immunofluorescence labelling were employed to assess the expression of *RelA*, SIRT1, SIRT6, SIRT7 and pro‐inflammatory factors. Dental pulp fibroblasts (DPFs) were transfected with siRNA to assess the biological role of SIRT7. A pulpitis model was utilized to evaluate the in vivo curative effect.

**Results:**

Preliminary results showed that RvE1 and LXA4 reduced the expression of *RelA* more markedly than other two SPMs. Both RvE1 and LXA4 treatment downregulated nuclear factor kappa B (NF‐κB) activation and increased the expression of SIRT1, SIRT6 and SIRT7, more so in combination than alone. Double‐immunofluorescence labelling showed that SIRT7 co‐localized with p‐p65 and Ac‐p65 in the nucleus. Inhibiting ChemR23 and ALX reversed the expression of *RelA* mRNA, p‐p65 and Ac‐p65 proteins, pro‐inflammatory factors, SIRT1, SIRT6 and SIRT7. Silencing *SIRT7* significantly increased p‐p65 and Ac‐p65 protein levels and decreased SIRT1 and SIRT6 expression. In vivo experiments showed that combined administration of RvE1 and LXA4 promoted pulpitis markedly to resolution.

**Conclusions:**

Combination of RvE1 and LXA4 effectively inhibited NF‐κB activation by upregulating *SIRT7* expression in DPFs, leading to reduced production of pro‐inflammatory factors and promotion of pulpitis resolution.

## Graphical abstract

Combination of resolvin E1 and lipoxin A4 could effectively upregulate the expression of SIRT7, inhibiting nuclear factor kappa B activation by dephosphorylation and deacetylation in dental pulp fibroblasts, and promote pulpitis resolution. Meanwhile, silencing SIRT7 may affect the expression of SIRT1 and SIRT6, indicating that they may synergistically promote inflammation resolution.

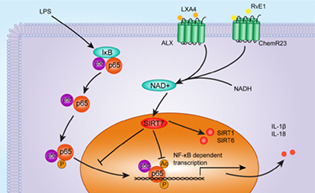

## 1 INTRODUCTION

Pulpitis is one of the most common inflammatory dental diseases, accounting for approximately 24%–44% of emergency problems observed in oral health clinics.^1^, ^2^ By causing irreversible damage of the dental pulp and periapical tissue, pulpitis eventually results in functional loss of the pulp and exfoliation of teeth.^3^ An inflammatory response is initiated after infection and/or injury as a protective mechanism to eliminate invading organisms and permit tissue repair. However, a dysregulated inflammatory response is the fundamental cause of chronic pulpitis and irreversible pulp damage.^4^ Hence, attenuation of excessive inflammation has been the most substantial challenge in modern endodontics.

Excessive inflammation is due to an imbalance between production and clearance of inflammatory mediators, which causes disruption of tissue homeostasis.^5^, ^6^ In contrast to the outdated view of a passive process of inflammatory dilution, resolution of inflammation is considered to be an active, programmed response that is ‘turned on’ in the body.^7^, ^8^ Pro‐resolution strategy is a relatively new approach for regulating inflammation, which avoids inhibition of the protective acute inflammatory response and reduces immune tolerance and other side effects of conventional anti‐inflammatory treatment.^9^, ^10^ The basis of resolution is sequestration of pro‐inflammatory cytokines, thereby improving the local inflammatory microenvironment, promoting clearance of apoptotic neutrophils and removal of inflammatory stimulation.^8^ Throughout the inflammatory process of pulpitis, an essential source of cytokines is the resident dental pulp fibroblasts (DPFs), which deteriorate the microenvironment, leading to soaring cytokine levels via a signal cascade.^11^, ^12^, ^13^ Therefore, regulation of DPFs could reduce the acute inflammatory infiltration and promote resolution.

Inflammatory resolution is mediated by specialized pro‐resolving mediators (SPMs), a family of bioactive lipids including, to date, four classes termed lipoxins, resolvins, maresins and protectins.^14^ In oral environment, independent evidence supports the protective effect of SPMs in experimental periodontitis. Lee et al.^15^ and Hasturk et al.^16^ demonstrated that resolvin E1 (RvE1) could decrease the expression of pro‐inflammatory genes and prevent alveolar bone loss by reducing osteoclast density and inflammatory cell infiltration. Besides, resolvin D2 has been shown to exhibit similar effects in murine periodontitis by restraining Th1 immunity.^17^ Furthermore, a recent clinical trial showed that methyl ester‐benzo‐lipoxin A4 (a lipoxin A4 analogue) reduced gingival inflammation and increased the abundance of pro‐resolution molecules that prevent periodontitis.^18^ These studies exhibit promise with respect to prevention and treatment of oral infectious inflammation. However, studies have rarely focused on the effects of SPMs in endodontics. Dondoni et al. first illustrated the protective role in a rat model of pulpitis.^19^ We previously showed that RvE1 could suppress inflammatory infiltration and accelerate pulp repair in pulpitis by reducing activation of nuclear factor kappa B (NF‐κB) in lipopolysaccharide (LPS)‐induced DPFs to some extent, but not to normal levels.^20^, ^21^ This is because SPMs are biosynthesized during resolution in a certain order and act on different stages and aspects of resolution.^22^, ^23^ Thus, complete inhibition of inflammatory pathway signalling and resolution cannot be achieved by using RvE1 alone, which is only synthesized in the later stage of resolution. To increase pulpitis resolution, combined application of different SPMs may be more effective. The optimal combination of SPMs and their mechanism of action in resolving pulpitis require further elucidation.

The key to sequestrating pro‐inflammatory factors is to inhibit the cascade of inflammatory pathway signalling.^24^ Among these pathways, the NF‐kB pathway is a major regulator of inflammation due to its ability to cause transcription of numerous genes involved in the inflammatory response.^25^, ^26^, ^27^ Therefore, inhibition of NF‐κB signalling in DPFs should be continued throughout the resolution process. Phosphorylation and nuclear localization, as well as steady binding to DNA through acetylation, are key steps in persistent NF‐κB activation and the inflammatory cascade. Persistent phosphorylation of NF‐κB is tightly regulated by acetylation, which controls the duration of NF‐kB activation.^28^ Deacetylation of NF‐kB has been demonstrated to be an intranuclear molecular switch that could prevent its activation by inhibiting the p65 subunit from entering the nucleus, as well as abrogating binding of activated p65 to DNA, and its translocation back to the cytoplasm.^29^ Thus, deacetylation may be a vital means to inhibit the NF‐kB pathway and promote resolution of pulpitis. It remains unknown whether combinations of SPMs could inhibit NF‐kB activation and promote its degradation by deacetylation.

Deacetylation of NF‐κB is directly regulated by sirtuins (SIRTs), a highly conservative family of deacetylases, including seven members in mammals. The enzymatic activities of SIRTs are amenable to regulation by nicotinamide adenine dinucleotide (NAD+), as NAD+ is a cofactor in the deacetylation reaction. SIRT1, SIRT6 and SIRT7 are predominantly expressed in the nucleus.^29^ As previous studies reported, SIRT1 and SIRT6 are able to deacetylate the p65 subunit, affecting its transcriptional activity and decreasing expression of pro‐inflammatory target genes.^30^, ^31^ It was recently suggested that SIRT7 is critical for inflammatory regulation and tissue homeostasis. Wyman et al. found that SIRT7 deficiency in primary pulmonary endothelial cells increased vascular permeability, which is involved in acute lung injury,^32^ although the underlying mechanism remains poorly understood. In infectious pulpitis, it also remains unknown whether a combination of SPMs could upregulate SIRT7 expression and promote resolution of inflammation. Besides, Fukuda et al. have demonstrated that SIRT7 cooperates with SIRT1 to promote OSX deacylation in bone metabolism.^33^ No data are available on the relationship of SIRT7 with SIRT1 or SIRT6 in NF‐kB deacetylation in an inflammatory environment.

In this study, we sought to clarify the mechanism of pulpitis resolution to provide new ideas and potential treatment strategies for vital pulp preservation. To this end, the inhibition of NF‐κB by four representative SPMs were first determined, which indicated the best combination of SPMs for further applications. Thereafter, we evaluated the effect of this combination of SPMs on LPS‐induced DPFs to investigate the phosphorylation and acetylation levels of NF‐kB and expression of SIRT1, SIRT6 and SIRT7, as well as that of pro‐inflammatory cytokines. Furthermore, the effect of SIRT7 on the RvE1 and lipoxin A4 (LXA4) combination was explored by silencing *SIRT7*, and the expression of SIRT1 and SIRT6 was observed concurrently to illustrate the relationship between them. Finally, an animal model of pulpitis (rat incisors) was employed to evaluate the therapeutic effect of the combined usage of RvvE1 and LXA4 on pulpitis.

## 2 MATERIALS AND METHODS

### 2.1 Cell culture

Ten‐third molars with healthy pulp, extracted for orthodontic requirements, were collected at the Affiliated Stomatology Hospital of Tongji University with approval from the Ethics Review Board (No. [2021]‐SR‐09). Briefly, the dental pulp was cut into fragments and digested by 2 mg/ml collagenase, type 1 (Sangon Biotech, Shanghai, China) for 1 h. After centrifugation at 1000 rpm for 10 min by an SL8 centrifuge (Thermo Scientific), the precipitate was removed into Dulbecco's modified Eagle's medium (Hyclone) with 10% of foetal bovine serum (Gibco), which was renewed every 3 days. Passage 3–5 DFPs were used for this study.

### 2.2 Cell stimulation

DPFs were seeded into six‐well plates with a density of 1 × 10^6^ per well and cultured overnight. For inflammatory stimulation, cells were stimulated with LPS (1 μg/ml, Sigma–Aldrich) for 3, 6, 12, 24, and 48 h, respectively, to detect p65 phosphorylation.

In the preliminary screening experiment, RvE1 alone (10 nM, Cayman Chemical Co.), LXA4 (10 nM, Cayman Chemical Co.), protectin D1 (PD1, 10 nM, Cayman Chemical Co.) and maresin‐1 (MaR1, 10 nM, Cayman Chemical Co.) were used to pretreat cells for 3 h. Then, cells were stimulated with LPS (1 μg/ml, Sigma–Aldrich) in the presence of the respective SPMs or vehicle for 24 h. In combined usage, cells were preincubated into RvE1 (10 nM, Cayman Chemical Co.), LXA4 (10 nM, Cayman Chemical Co.) and RvE1 + LXA4, respectively, for 3 h. Cells were subsequently stimulated with 1 μg/ml of LPS for 24 h.

For ChemR23 (RvE1 receptor) and/or ALX (LXA4 receptor, also called FPR2) inhibition, cells were pretreated with *N*,*N*,*N*‐trimethyl‐3‐(1‐naphthyl)‐3‐oxopropan‐1‐aminium iodide (α‐NETA, 1 μM, Cayman Chemical Co.) and/or N‐Boc‐Phe‐Leu‐Phe‐Leu‐Phe (BOC‐2, 1 μM, GenScript) for 1 h before RvE1 and/or LXA4 administration.

To silence *SIRT7*, cells were transfected with 50‐nM small interfering RNA (si‐*SIRT7*, synthesized by RiboBio) using Lipofectamine 3000 (Invitrogen) in Opti‐MEM (Gibco). Transfection with Lipofectamine 3000 without si‐*SIRT7* was used as a mock control. At 24 h after transfection, cells were employed for subsequent stimulation. The si‐*SIRT7* sequence is listed in Table 1.

**TABLE 1:** Primers used for the selected human genes

Target	Sequences (5′ → 3′)	Purpose
*RelA*	Forward: GGACATGGACTTCTCAGCCC	qPCR
	Reverse: CACAAAGTTGGGGGCAGTTG	qPCR
*NLRP3*	Forward: GATCTTCGCTGCGATCAACAG	qPCR
	Reverse: CGTGCATTATCTGAACCCCAC	qPCR
*caspase1*	Forward: TTTCCGCAAGGTTCGATTTTCA	qPCR
	Reverse: GGCATCTGCGCTCTACCATC	qPCR
*IL‐1β*	Forward: GGATCTCCTGTCCATCAGCC	qPCR
	Reverse: GGAGCGAATGACAGAGGGTT	qPCR
*IL‐18*	Forward: TCTTCATTGACCAAGGAAATCGG	qPCR
	Reverse: TCCGGGGTGCATTATCTCTAC	qPCR
*IL‐6*	Forward: TGCAATAACCACCCCTGACC	qPCR
	Reverse: GTGCCCATGCTACATTTGCC	qPCR
*TNF‐α*	Forward: CACAGTGAAGTGCTGGCAAC	qPCR
	Reverse: AGGAAGGCCTAAGGTCCACT	qPCR
*CCL2*	Forward: TCAAACTGAAGCTCGCACTCT	qPCR
	Reverse: GGGGCATTGATTGCATCTGG	qPCR
*CCL7*	Forward: AGAAGGACCACCAGTAGCCA	qPCR
	Reverse: CCACTTCTGTGTGGGGTCAG	qPCR
*SIRT1*	Forward: TAGCCTTGTCAGATAAGGAAGGA	qPCR
	Reverse: ACAGCTTCACAGTCAACTTTGT	qPCR
*SIRT6*	Forward: CAAGTGTAAGACGCAGTACGT	qPCR
	Reverse: ATGTACCCAGCGTGATGGAC	qPCR
*SIRT7*	Forward: ATGAGCAGAAGCTGGTGC	qPCR
	Reverse: CTGTCTGGTGTCTGTGGA	qPCR
*ALX*	Forward: TCTTGCTCTAGTCCTTACCTTGC	qPCR
	Reverse: AATGACAAACCGGATAATCCCTC	qPCR
*ChemR23*	Forward: TGGACTACCACTGGGTTTTCG	qPCR
	Reverse: CGAGAGATGGGGAACTCAAGAAG	qPCR
*β‐Actin*	Forward: CATGTACGTTGCTATCCAGGC	qPCR
	Reverse: CTCCTTAATGTCACGCACGAT	qPCR
si‐*SIRT7*	CUCACCGUAUUUCUACUACUA	siRNA

Abbreviation: qPCR, quantitative polymerase chain reaction.

### 2.3 Real‐time quantitative PCR

Total cellular RNA was extracted using a TRIeasy Total RNA Extraction Reagent (Yeasen) for reverse‐transcription, using a cDNA Synthesis SuperMix for quantitative polymerase chain reaction (qPCR) with gDNA Eraser (Yeasen). To quantify mRNA levels, real‐time qPCR was performed using a qPCR SYBR Green Master Mix (Yeasen) on a LightCycler®96 (Roche) device. The primer sequences are listed in Table 1. Data were normalized to the housekeeping gene *ACTB* (β‐actin), and relative expression was evaluated using the 2^−ΔΔ*Ct*^ method.

### 2.4 Western blotting

For total protein extraction, cells were lysed with Radio Immunoprecipitation Assay (RIPA, Beyotime Biotechnology) containing 1 mM of phenylmethanesulfonyl fluoride (Beyotime), 1 × phosphatase inhibitor cocktail and protease inhibitor cocktail (Beyotime). Isolation of cytosolic and nuclear protein fractions were achieved using nuclear and cytoplasmic protein extraction kits (Solarbio). Cell lysates were cleared by centrifuging at 12,000 rpm at 4°C using a high‐speed refrigerated centrifuge (LX‐165T, Haier Biomedical) for 15 min.

During western blotting, 20 μg of the supernatants were separated by dodecyl sulfate, sodium salt (SDS)‐polyacrylamide gel electrophoresis. Subsequently, the proteins were transferred to a nitrocellulose (NC) membrane (Biosharp Corp). The membranes were blocked with 5% of bovine serum albumin (BSA; Sangon Biotech) in Tris‐buffered saline containing 0.1% of Tween‐20 for 1 h at room temperature before incubation with the primary and secondary antibody. The dilution of all primary antibodies was 1:1000, while that of secondary antibodies was 1:5000. The antibodies used include rabbit anti‐phospho‐p65 (#AF2006, Affinity Biosciences), rabbit anti‐acetyl‐p65 (#AF1017, Affinity), rabbit anti‐p65 (#AF5006, Affinity), rabbit anti‐SIRT1 (#DF6033, Affinity), rabbit anti‐SIRT6 (#DF12739, Affinity), rabbit anti‐SIRT7 (#DF6161, Affinity), rabbit anti‐NLRP3 (#DF7438, Affinity), rabbit anti‐caspase1 (#AF5418, Affinity), rabbit anti‐IL‐1β (#AF5103, Affinity), rabbit anti‐IL‐18 (#DF6252, Affinity), mouse anti‐β‐tubulin (#T0023, Affinity), mouse anti‐β‐actin (#T0022, Affinity), mouse anti‐PCNA (#BF0704, Affinity), anti‐rabbit IgG HRP (#S0001, Affinity) and anti‐mouse IgG HRP (#S0002, Affinity). Thereafter, the bands were detected by using an Amersham Imager 680 apparatus (Cytiva).

### 2.5 Double‐immunofluorescence labelling

Cells cultured on coverslips were fixed in 4% of paraformaldehyde (Sangon Biotech) for 20 min, immersed in 0.1% of triton X‐100 (Sangon Biotech) for 15 min, followed by blocking in 5% of BSA for 1 h at room temperature. The combination of mouse anti‐SIRT7 (#sc‐365344, Santa Cruz) and rabbit anti‐phospho‐p65 (or rabbit anti‐acetyl‐p65) antibodies were employed concurrently to observe the co‐location of SIRT7 and phospho‐p65 (or acetyl‐p65), while mouse anti‐phospho‐p65 (#sc‐136548, Santa Cruz) and rabbit anti‐acetyl‐p65 were used for co‐labelling of phospho‐p65 and acetyl‐p65, respectively. All primary antibodies were used at 1:2000 dilutions. These primary antibodies were detected using Alexa Fluor 546 goat anti‐rabbit IgG (H + L) (Invitrogen) and Alexa Fluor 488 goat anti‐mouse IgG (H + L) (Invitrogen, used at 1:2000 dilutions) secondary antibodies. Nuclei were stained with 4′,6‐diamidino‐2‐phenylindole (DAPI, Sigma–Aldrich). Images of the cells were obtained using a Nikon A1R microscope (Nikon, Minato City, Japan) with a fluorescence light source and filters.

### 2.6 NAD+/NADH assay

Intracellular NAD+ and NADH levels were determined by means of an NAD+/NADH assay kit (WST‐8 method, Beyotime Biotechnology) according to the manufacturer's instructions. Briefly, 200 μl of cold lysis buffer was used to lyse cells. Twenty microliters of supernatant were added into a 96‐well plate to measure total NAD, and samples were incubated at 60°C for 30 min for NADH measurement. Subsequently, 90 μl of alcohol dehydrogenase was added, and the samples were incubated at 37°C for 10 min. Finally, 10 μl of chromogenic solution was used for colour generation. A standard curve was established at the same time. The amount of NAD or NADH was measured using a Synergy H1 Hybrid Multi‐Mode Microplate Reader (BioTek Instruments) at 450‐nm wavelength, and the content of NAD+ was determined by subtracting NADH from the total NAD. Data are shown as the NAD+/NADH ratio.

### 2.7 Enzyme‐linked immunosorbent assay

We centrifuged media in which cells had been cultured for 72 h at 12,000 rpm at 4°C for 10 min to remove debris. The supernatants were collected for enzyme‐linked immunosorbent assays (ELISAs) using human IL‐1β and IL‐18 ELISA kits (R&D Systems) according to the manufacturer's instruction.

### 2.8 Animal experiment

Eight‐week‐old male Sprague–Dawley rats (weighing 200–250 g, purchased from Shanghai Jiesijie Laboratory Animal Co., Ltd), without any dental decay, were used for an in vivo experiment. All animal experiments were approved by the Ethics Review Board of the Affiliated Stomatology Hospital of Tongji University (NO. [2021]‐DW‐21). The animal model was established similar to our previous study.^20^ Briefly, under general anaesthesia using 1% Pelltobarbitalum Natricum (Sangon Biotech), 2 mm of the crowns of the maxillary incisors were gently ground using a no. 1/2 round burr (Mani Inc), and access to the pulp was gained using a #20 K‐file followed by enlargement using with a #40 K‐file (Mani). Pulp was exposed to the oral environment for 12 h. Thereafter, 0.9% of sterile saline solution was used for irrigation to remove debris above the pulp. The cavities were dried with sterile cotton pellets and were covered using sponge (Jinling Pharmaceutical Co., Ltd) with 50‐ng RvE1 and/or LXA4. A control group was set up with a sham, non‐exposure operation. Rats were euthanized to extract samples at days 1, 3 and 7.

### 2.9 Histology and immunohistochemistry

Samples were fixed in 4% of paraformaldehyde for 24 h, decalcified in 12% of EDTA (Sangon Biotech) for 10 weeks and embedded in paraffin (Sangon Biotech). Serial sections (4‐μm‐thick) were stained with haematoxylin–eosin (HE; Sangon Biotech) and histological analysis was conducted.

To verify infiltration of immune cells, immunofluorescence of CD11b (#DF2911, Affinity, used at 1:200 dilutions) was applied. Immunohistochemical staining was performed as previously described.^20^ Anti‐NLRP3, anti‐caspase1, anti‐IL‐1β, anti‐IL‐18 (mentioned above) and anti‐OCN (PB1009, BOSTER) antibodies were used as primary antibodies at 1:200 dilutions. Double‐immunofluorescence labelling was also performed as described above.

### 2.10 Statistical analysis

Three replicates were performed in all experiments independently, and data were expressed as mean ± standard deviation (SD). Statistical analysis was performed using SPSS 22.0 software (IBM SPSS Inc.) via one‐way analysis of variance and Tukey's post hoc test. Statistical significance was indicated by *p* < 0.05.

## 3 RESULTS

### 3.1 Combined administration of RvE1 and LXA4 downregulates *RelA* mRNA levels and production of inflammatory cytokines by LPS‐induced DPFs

**FIGURE 1:**
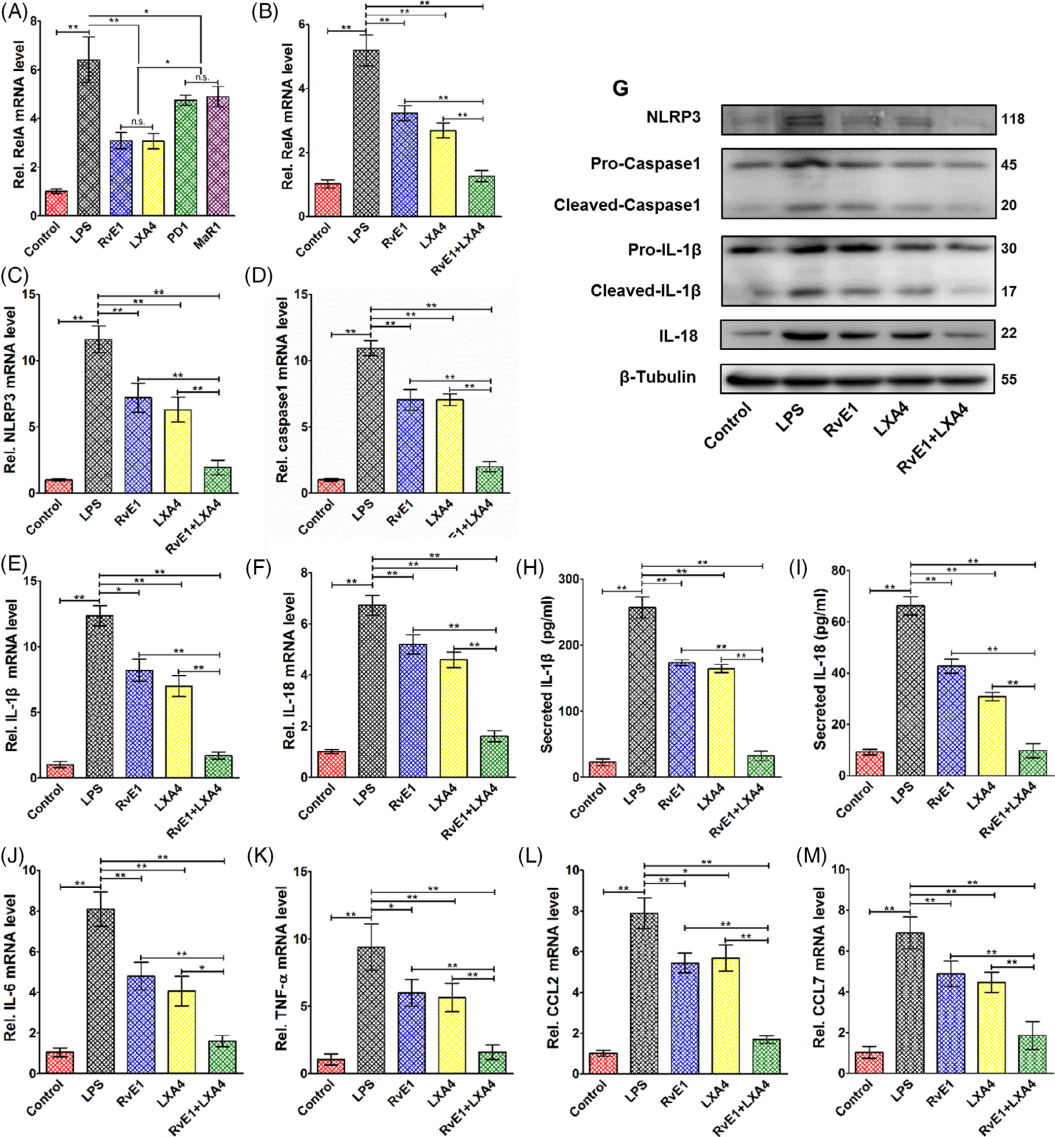
Effects of combined administration of RvE1 and LXA4 on pro‐inflammatory factor expression. (A) Expression of *RelA* mRNA in LPS‐induced DPFs after treatment with four representative specialized pro‐resolving mediators. (B) Expression of *RelA* mRNA in LPS‐induced DPFs after treatment with RvE1 or LXA4 alone, and a combination of RvE1 and LXA4. (C) *NLRP3*, (D) *caspase‐1*, (E) *IL‐1β* and (F) *IL‐18* mRNA levels on LPS‐induced DPFs detected by qPCR and (G) their protein levels tested by western blotting (normalized to that of β‐tubulin). (H) IL‐1β and (I) IL‐18 protein levels from cultural supernatant detected by ELISA. Other NF‐κB‐dependent expression of genes (J) *IL‐6*, (K) *TNF‐α*, (L) *CCL2* and (M) *CCL7* detected by qPCR. (*p < 0.05 and **p < 0.01). DPFs, dental pulp fibroblasts; ELISA, enzyme‐linked immunosorbent assay; LPS, lipopolysaccharide; LXA4, lipoxin A4; NF‐κB, nuclear factor kappa B; qPCR, quantitative polymerase chain reaction; RvE1, resolvin E1

Four representative SPMs were used in LPS‐induced DPFs. Preliminary results showed that RvE1 and LXA4 reduced the expression of *RelA* more markedly than did PD1 and MaR1 (Figure 1A). Moreover, both RvE1 and LXA4 showed the best inhibitory effect on *RelA* expression at the concentration of 10 nM (Figure S1A,B), which was applied in follow‐up experiments. Then, a combination of RvE1 and LXA4 was used, which resulted in a significantly greater decrease in *RelA* expression than that achieved with RvE1 or LXA4 alone (Figure 1B).

For downstream inflammatory cytokines, qPCR results demonstrated that combined administration of RvE1 and LXA4 could lower the mRNA levels of *NLRP3*, *caspase‐1*, *IL‐1β*, and *IL‐18*, which are related to NLRP3 inflammasomes and whose transcription is regulated by NF‐κB, in LPS‐induced DPFs, more significantly than could RvE1 or LXA4 alone (Figure 1C–F). Furthermore, their protein expression levels detected by western blotting (Figure 1G) and the amount of secreted IL‐1β and IL‐18 (Figure 1H,I) detected by ELISA showed a similar trend. Moreover, the combination of RvE1 and LXA4 significantly reduced the NF‐κB‐dependent transcription of other genes, including *IL‐6, TNF‐α, CCL2* and *CCL7* (Figure 1J–M).

### 3.2 Combined administration of RvE1 and LXA4 promotes expression of SIRTs and suppresses the phosphorylation and acetylation of NF‐κB in LPS‐induced DPFs

**FIGURE 2:**
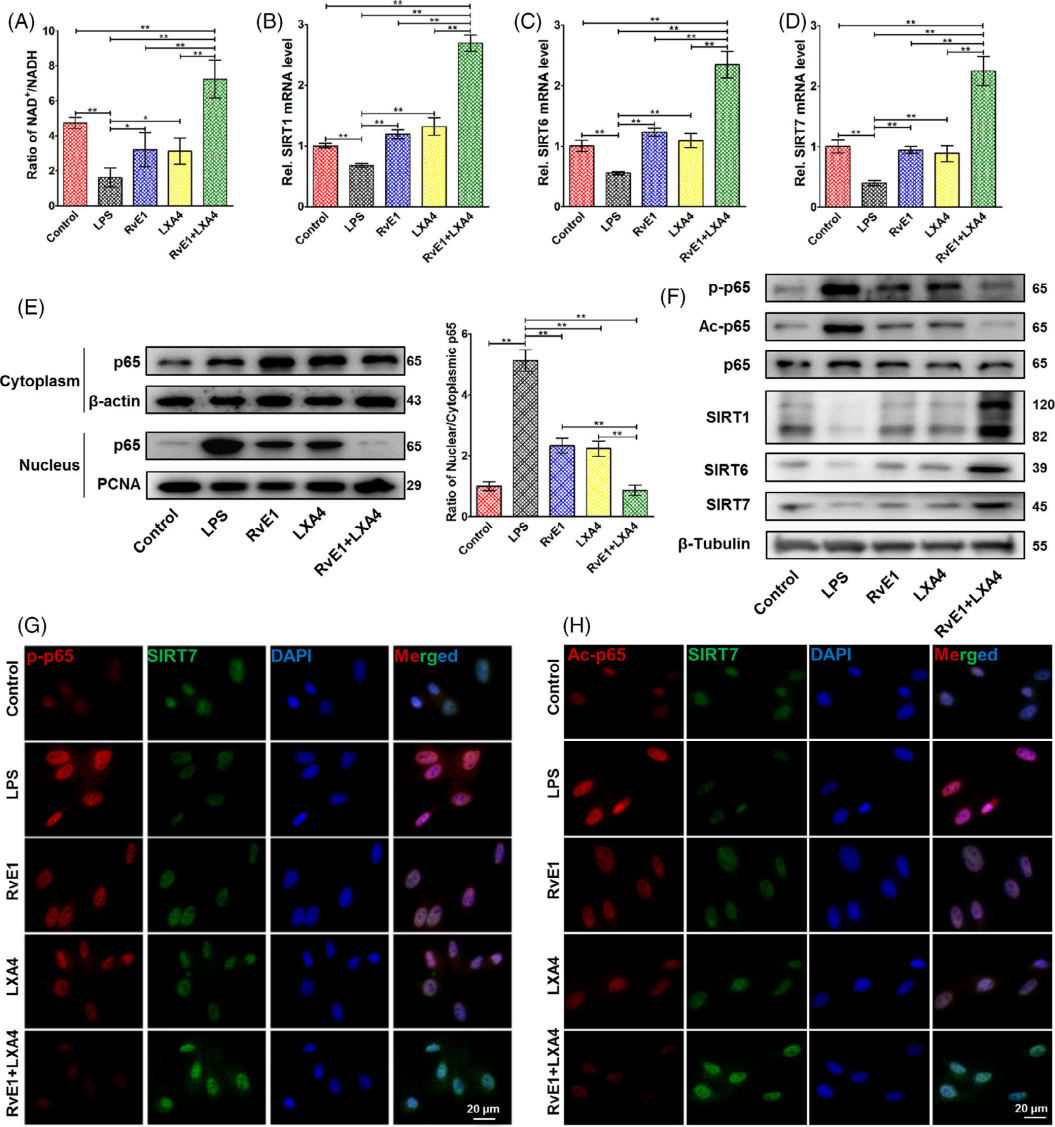
Effects of the RvE1 and LXA4 combination on NF‐κB modification. (A) NAD+/NADH ratio measured by a NAD+/NADH assay kit. (B) *SIRT1*, (C) *SIRT6*, and (D) *SIRT7* mRNA levels detected by qPCR. (E) Expression of p65 protein in both cytoplasm and nucleus detected by western blotting, normalized to β‐actin (in cytoplasm) or PCNA (in nucleus). (F) Phosphorylation and acetylation levels of p65 and expressions of SIRT1, SIRT6 and SIRT7 protein (normalized to that of β‐tubulin) detected by western blotting. (G) Representative double‐immunofluorescence labelling images of SIRT7 (green) and p‐p65 (red), and the nuclei were stained with DAPI (blue). (H) Representative double‐immunofluorescence labelling images of SIRT7 (green) and Ac‐p65 (red), and the nuclei were stained with DAPI (blue). (*p < 0.05 and **p < 0.01). DAPI, 4′,6‐diamidino‐2‐phenylindole; LXA4, lipoxin A4; NAD+, nicotinamide adenine dinucleotide; NF‐κB, nuclear factor kappa B; qPCR, quantitative polymerase chain reaction; RvE1, resolvin E1

Combined administration of RvE1 and LXA4 strongly increased the NAD+/NADH ratio, which was decreased by LPS, more markedly than by either treatment alone, even in normal‐cultured DPFs (Figure 2A). RvE1 and LXA4 alone increased the mRNA levels of *SIRT1*, *SIRT6* and *SIRT7* to approximately the expression level of the control group, while their combined use elevated their expression to a much higher level (Figure 2B–D). Western blotting results showed a similar tendency in terms of protein expression (Figure 2F). The time‐dependent pattern of p65 phosphorylation was pre‐investigated in LPS‐induced DPFs. As shown in Figure S1C, p65 phosphorylation was strongly increased 3 h after LPS stimulation and declined at 6 h. Subsequently, p65 phosphorylation peaked at 12 and 24 h after LPS stimulation and then decreased after 48 h of incubation. We incubated DPFs with LPS for 24 h in follow‐up experiments.

Translocation of p65 was detected by western blotting of nuclear and cytosolic protein, respectively. As shown in Figure 2E, the amount of nuclear p65 protein was sharply augmented in LPS‐induced DPFs, while cytoplasmic p65 protein only slightly increased, resulting in an increased ratio of nuclear to cytoplasmic p65. After RvE1‐ or LXA4‐only treatment, the amount of nuclear p65 protein decreased, with a small increase in cytoplasmic p65. The combination of RvE1 and LXA4 caused a further decline of nuclear p65 and a slight decrease in cytoplasmic p65. Meanwhile, the phosphorylation and the acetylation of p65 (p‐p65 and Ac‐p65) were concurrently decreased, and combined usage showed a much greater inhibition than using separately (Figure 2F).

Double‐immunofluorescence labelling was employed to observe the cellular co‐localization of SIRT7 and p‐p65 (Figure 2G) or Ac‐p65 (Figure 2H). Images revealed that after LPS stimulation, both p‐p65 and Ac‐p65 were highly expressed in and around the nucleus, while SIRT7 maintained a low expression in the nucleus. In contrast, increased SIRT7 expression and reduced p‐p65 and Ac‐p65 levels were observed upon combined administration of RvE1 and LXA4. These effects were more marked for the combined treatment than for either single treatment.

### 3.3 Inhibition of ChemR23 and ALX reverses the inhibitory effect of RvE1 and LXA4 on NF‐κB

**FIGURE 3:**
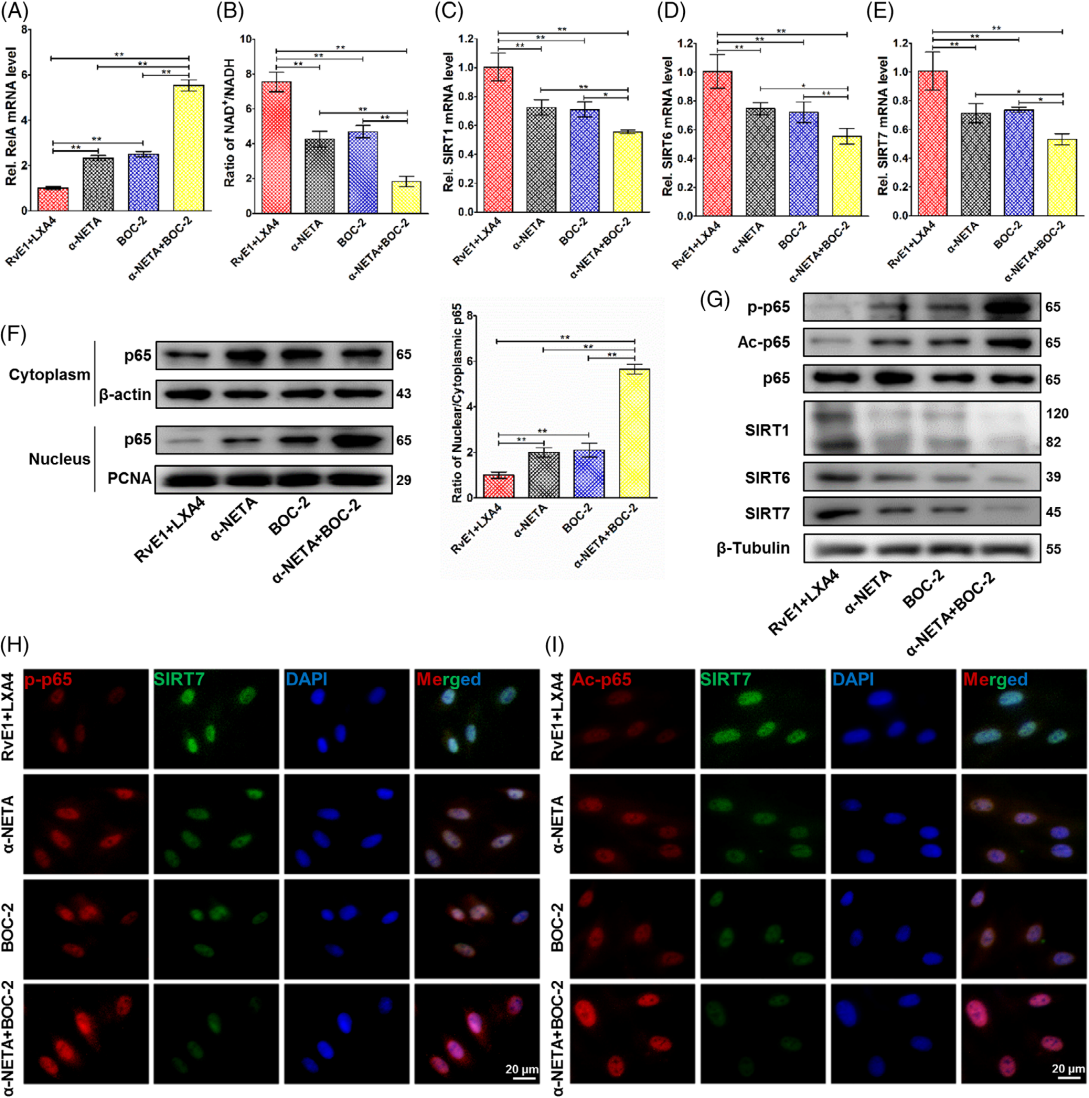
Pro‐resolving effect of the RvE1 and LXA4 combination after ChemR23 and/or ALX inhibition. (A) Expression of *RelA* mRNA detected by qPCR. (B) NAD+/NADH ratio measured by an NAD+/NADH assay kit. (C) *SIRT1*, (D) *SIRT6* and (E) *SIRT7* mRNA levels detected by qPCR. (F) Expression of p65 protein in both cytoplasm and nucleus detected by western blotting, normalized to β‐actin (in cytoplasm) or PCNA (in nucleus). (G) Phosphorylation and acetylation levels of p65 and expressions of SIRT1, SIRT6 and SIRT7 protein (normalized to β‐tubulin) detected by western blotting. (H) Representative double‐immunofluorescence labelling images of SIRT7 (green) and p‐p65 (red), and the nuclei were stained with DAPI (blue). (I) Representative double‐immunofluorescence labelling images of SIRT7 (green) and Ac‐p65 (red), and the nuclei were stained with DAPI (blue). (*p < 0.05 and **p < 0.01). DAPI, 4′,6‐diamidino‐2‐phenylindole; LXA4, lipoxin A4; NAD+, nicotinamide adenine dinucleotide; qPCR, quantitative polymerase chain reaction; RvE1, resolvin E1

To verify the synergy of RvE1 and LXA4 on NF‐κB inhibition, their receptor antagonists, α‐NETA and BOC‐2, respectively, were used to block ChemR23 (RvE1 receptor in DPFs) and ALX (LXA4 receptor), singly or in combination. Data from qPCR indicated that the antagonistic efficiency of ALX was around 60% (Figure S2A), while that of ChemR23 was about 80% (Figure S2B). Through ChemR23 or ALX inhibition, the suppressive effect of RvE1 and LXA4 on NF‐κB was significantly discounted, and the simultaneous inhibition abrogated the decrease in *RelA* mRNA (Figure 3A). The NAD+/NADH ratio (Figure 3B) and the expression of *SIRT1*, *SIRT6* and *SIRT7* (Figure 3C–E) were also decreased. By concurrent antagonizing, the effects of RvE1 and LXA4 were almost fully reversed (Figure 3B–E). At the protein level, western blotting results showed that both α‐NETA and BOC‐2 initiated an increasing proportion of nuclear p65, with a mild increase in cytoplasmic p65, thus the ratio of nuclear to cytoplasmic p65 increased. Co‐inhibition of ChemR23 and ALX resulted in a larger increase in nuclear p65 (Figure 3F). Moreover, p‐p65 and Ac‐p65 were highly expressed when ChemR23 and/or ALX were blocked (Figure 3G), which showed a similar trend as seen in the mRNA. Representative images of double‐immunofluorescence labelling displayed reduced expression of SIRT7 and increased expression of p‐p65 and Ac‐p65 (Figure 3H,I).

Downstream from NF‐κB, the mRNA levels of *NLRP3*, *caspase‐1*, *IL‐1β* and *IL‐18* showed a similar tendency as the *RelA* mRNA level. When the receptor antagonists were used alone or together, the inhibitory effect of the RvE1 and LXA4 combination was weakened (Figure S2C–F). At the protein level, western blotting (Figure S2G) and ELISA (Figure S2H,I) results showed that their expression levels and the levels of secreted IL‐1β and IL‐18 were significantly increased upon inhibition of ChemR23 and ALX.

### 3.4 Silencing *SIRT7* weakens suppression of NF‐κB by the RvE1 and LXA4 combination

**FIGURE 4:**
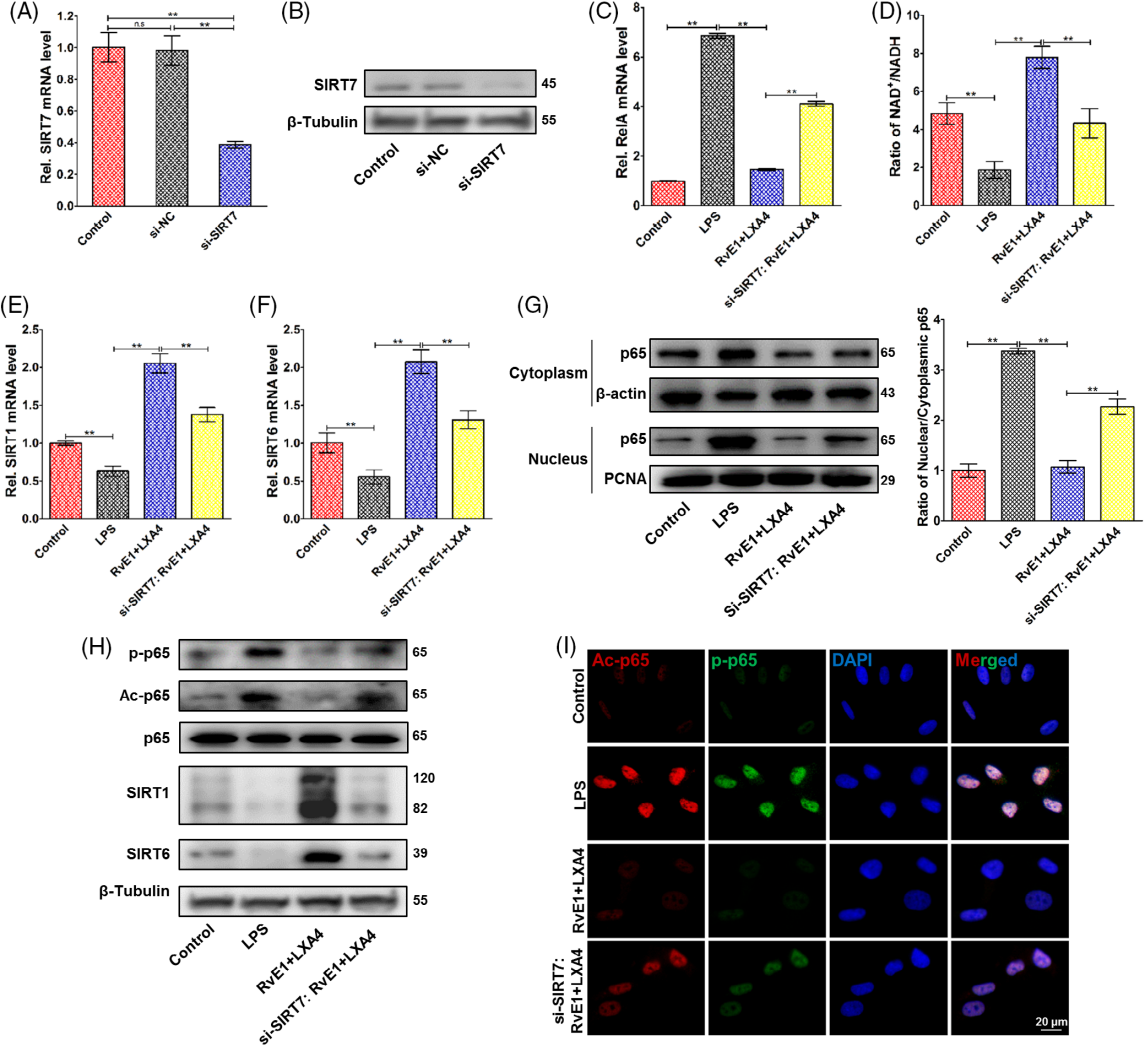
Pro‐resolving effect of the RvE1 and LXA4 combination after *SIRT7* silencing. (A) Silencing efficiency of si‐*SIRT7* on mRNA level, and (B) that of in protein level. (C) Expression of *RelA* mRNA detected by qPCR. (D) NAD+/NADH ratio measured by an NAD+/NADH assay kit. (E) *SIRT1* and (F) *SIRT6* mRNA levels detected by qPCR. (G) Expression of p65 protein in both cytoplasm and nucleus detected by western blotting, normalized to β‐actin (in cytoplasm) or PCNA (in nucleus). (H) Phosphorylation and acetylation levels of p65 and expressions of SIRT1, SIRT6 and SIRT7 protein (normalized to β‐tubulin) detected by western blotting. (I) Representative double‐immunofluorescence labelling images of p‐p65 (green) and Ac‐p65 (red), and the nuclei were stained with DAPI (blue). (*p < 0.05 and **p < 0.01). DAPI, 4′,6‐diamidino‐2‐phenylindole; LXA4, lipoxin A4; NAD+, nicotinamide adenine dinucleotide; qPCR, quantitative polymerase chain reaction; RvE1, resolvin E1

To assess the biological role of SIRT7 in the resolution of pulpitis, siRNA targeting *SIRT7* was designed and transfected into DPFs. Results of qPCR (Figure 4A) and western blotting (Figure 4B) revealed a silencing efficiency exceeding 60%, making it suitable for subsequent experiments. *SIRT7*‐knockdown abrogated the NF‐κB‐inhibitory effect of RvE1 and LXA4 in LPS‐induced DPFs, as the expression of *RelA* mRNA significantly increased (Figure 4C). Interestingly, the NAD+/NADH ratio showed a reduction in feedback (Figure 4D), and the expression of *SIRT1* and *SIRT6* was also decreased (Figure 4E,F). Western blotting results displayed a similar trend for SIRT1 and SIRT6 protein levels (Figure 4H). In addition, SIRT7 was also involved in the activation of NF‐κB p65. Silencing SIRT7 partially reversed the inhibition of RvE1 and LXA4 combination on nuclear translocation of p65 (Figure 4G); moreover, western blotting (Figure 4H) and double‐immunofluorescence labelling (Figure 4I) suggested that *SIRT7*‐knockdown significantly increased p65 phosphorylation and acetylation after RvE1 and LXA4 treatment of LPS‐induced DPFs, indicating increased activation of NF‐κB.

### 3.5 Combined treatment with RvE1 and LXA4 promotes resolution of rat pulpitis

**FIGURE 5:**
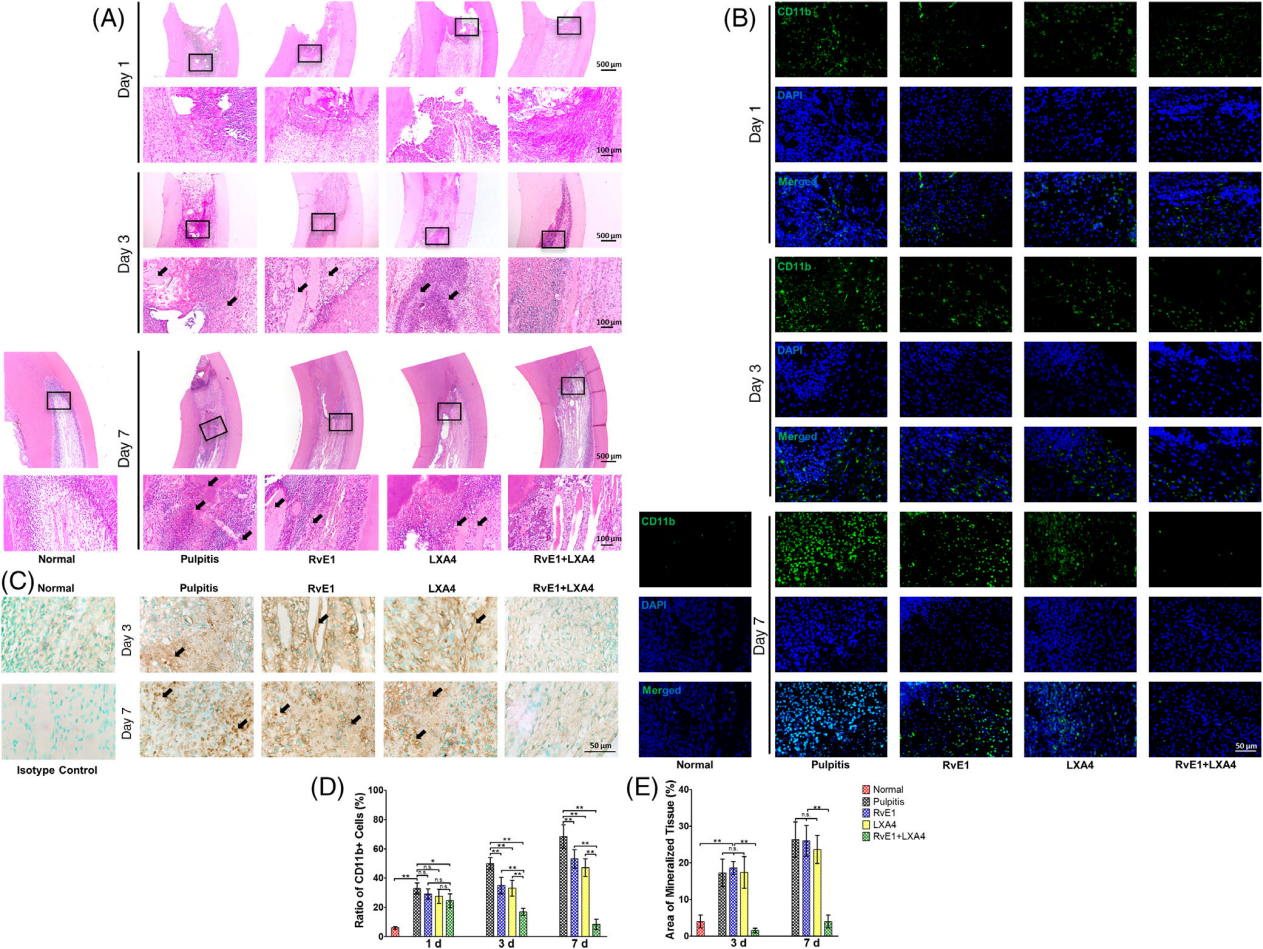
Therapeutic effect of combined administration of RvE1 and LXA4 in a rat pulpitis model. (A) Representative haematoxylin–eosin‐stained images on days 1, 3 and 7 after surgery. (B) Representative immunofluorescence images of CD11b on days 1, 3 and 7 after surgery. (C) Representative immunohistochemistry‐stained images of OCN on days 3 and 7 after surgery. (D) Statistical analysis of number of immune cells. (E) Statistical analysis of the area of mineralized tissue. (*p < 0.05 and **p < 0.01). LXA4, lipoxin A4; RvE1, resolvin E1

A pulpitis model in rat incisors was utilized to evaluate the in vivo curative effect of the RvE1 and LXA4 combination. Images of HE staining (Figure 5A) and CD11b immunofluorescence (Figure 5B) suggested that infiltration of immune cells was significantly less in the RvE1‐ and/or LXA4‐treated group than in the pulpitis tissue at day 1. On day 3, the inflammation continued to spread in the pulpitis tissue but was notably limited in the RvE1‐ and/or LXA4‐treated group (Figure 5D). In the RvE1‐ and LXA4‐only treatment groups, ectopic mineralization in the pulp cavity, a typical feature of the chronic pulpitis stage, was markedly present, while it was virtually absent in rats treated with the combination of RvE1 and LXA4 (Figure 5A). Immunohistochemical staining showed that ectopic mineralization was positively expressed of OCN. A large area of positive signals was observed in pulpitis and RvE1‐ or LXA4‐only treatment groups, while there were few positive signals after combination treatment with RvE1 and LXA4 (Figure 5C). On day 7, inflammatory infiltration was widespread, and the pulp opening remained unclosed in the pulpitis group, while there was also a large amount of ectopic mineralization with high OCN expression in the pulp cavity, similar to the RvE1‐ or LXA4‐only treatment groups. Inversely, the pulp in the rats that received combination treatment with RvE1 and LXA4 showed no inflammatory infiltration and very little mineralized tissue near the dentin, which approximated normal pulp tissue (Figure 5A–E).

**FIGURE 6:**
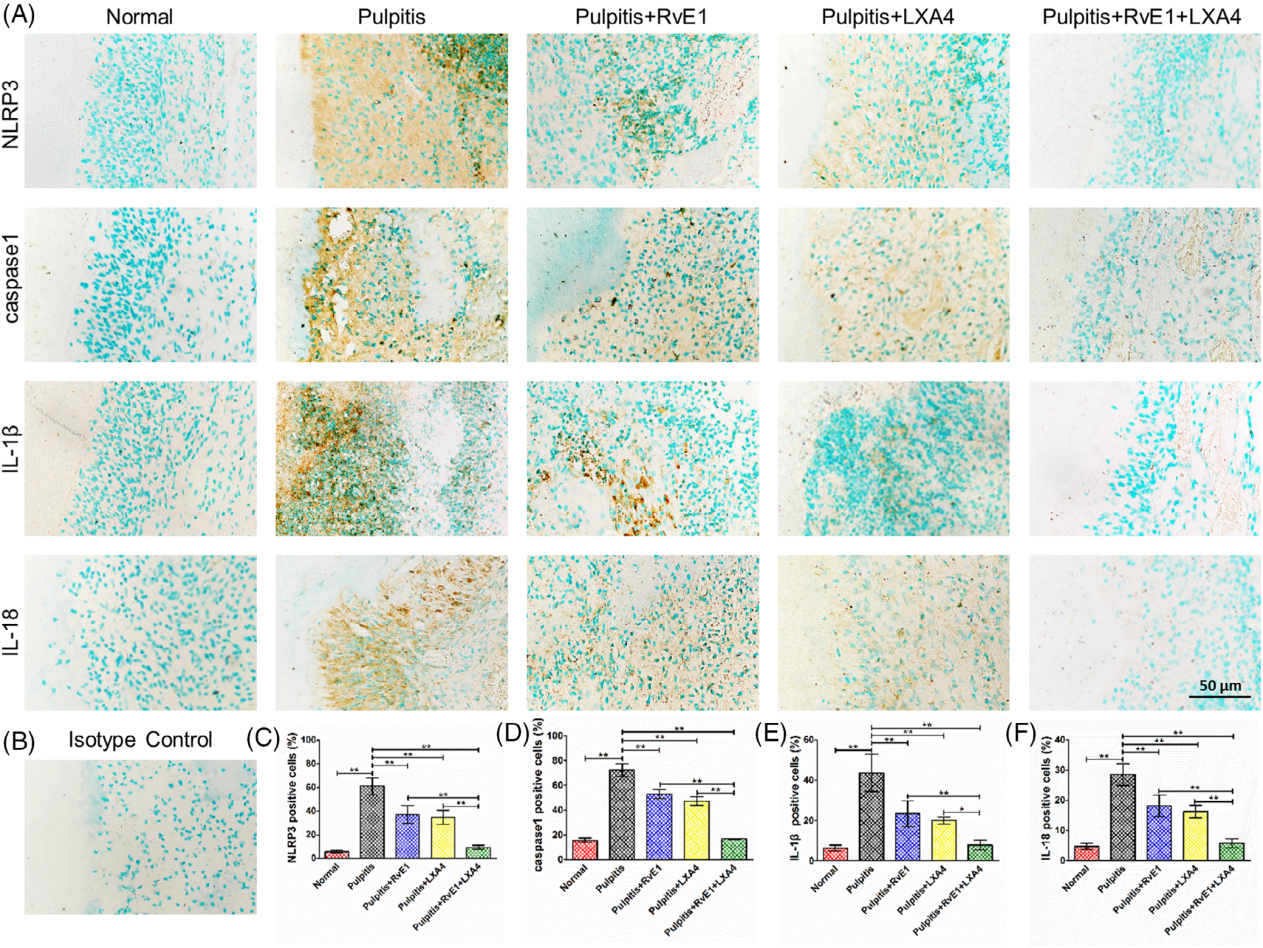
(A) Representative immunohistochemistry‐stained images of NLRP3, caspase‐1, IL‐1β, and IL‐18 of day 3 samples. (B) Isotype control. Statistical analysis of (C) NLRP3‐, (D) caspase‐1‐, (E) IL‐1β‐ and (F) IL‐18‐positive cells. (*p < 0.05 and **p < 0.01)

Images of double‐immunofluorescence labelling of day 3 samples indicated a similar tendency to the in vitro experiments. In the pulpitis group, both p‐p65 and Ac‐p65 were expressed at higher levels, while SIRT7 showed a lower expression than in normal pulp. After combined RvE1 and LXA4 treatment, SIRT7 increased and p‐p65 and Ac‐p65 decreased, with changes that were more significant than with either treatment alone (Figure S3A,B). Immunohistochemistry staining for NLRP3, caspase‐1, IL‐1β and IL‐18 (Figure 6A,B) and statistical analysis (Figure 6C–F) showed the same trend, indicating that use of RvE1 or LXA4 alone could reduce the expression of these inflammatory markers to some extent, but that their levels were still higher than in normal pulp tissue. However, combined administration of RvE1 and LXA4 could promote pulpitis resolution to a greater extent, facilitating recovery of homeostasis in the pulp tissue.

## 4 DISCUSSION

In this study, we investigated whether combined administration of RvE1 and LXA4 could promote resolution of pulpitis better than RvE1 or LXA4 alone. We also investigated the associated mechanism in DPFs and in a rat incisor model of pulpitis. We showed that combined administration of RvE1 and LXA4 could effectively promote pulpitis resolution by inhibiting NF‐κB activation via upregulation of SIRT7 expression, reducing expression of pro‐inflammatory factors.

Inflammatory regulation is crucial to vital pulp preservation and may be better achieved by pro‐resolving than by traditional anti‐inflammatory approaches. In pulp tissue, a variety of cells are involved in inflammatory resolution, which is directly regulated by SPMs. It has been widely demonstrated that SPMs can limit neutrophil infiltration, promote their apoptosis and stimulate macrophage efferocytosis in disorders such as atherosclerosis, intestinal epithelial injure and dry eye disease.^7^, ^34^, ^35^, ^36^ Except for the immune cells, resident cells of the pulp, including DPFs, stem cells and odontoblasts, contribute substantially to inflammatory resolution and are critical for tissue homeostasis.^37^ Horibe et al. found that ALX could be observed in odontoblastic layer during physiological and reparative dentin formation.^38^ Our group has previously shown that RvE1 can accelerate repair of injured pulp by regulating inflammation and promoting odontogenic differentiation in dental pulp stem cells.^21^ These results highlight the pro‐regeneration function of SPMs in dental pulp, but gaps remain regarding understanding of inflammatory regulation. Our previous study also verified that using RvE1 alone could inhibit inflammatory reactions and NF‐κB activation to a certain extent but failed to achieve complete resolution of pulpitis.^20^ In our present study, we also found that single usage of RvE1 or LXA4 significantly reduced the expression of pro‐inflammatory cytokines (Figure 1B) but failed to decrease to that of normal pulp. These results were similar to previous findings regarding SPMs in periodontitis^15^ and lung injury.^39^ SPMs act at different stages of resolution and on diverse inflammatory biomarkers.^22^ Therefore, combined administration of SPMs may be an optimal tactic in pro‐resolving approach.

SPMs are a family of bioactive lipids derived from ω‐3 and ω‐6 essential polyunsaturated fatty acids including, to date, four classes named lipoxins, resolvins, maresins and protectins.^14^ Considering the classification, metabolic precursors and receptors of SPMs, we selected one from each category for preliminarily screening. We found that LXA4 and RvE1 showed a greater inhibitory effect on NF‐κB signalling in LPS‐induced DPFs than the two other SPMs (Figure 1A). Hence, we applied them in combination in the follow‐up experiments. In this study, we first explored the optimal concentration of RvE1 and LXA4 on LPS‐induced DPFs, finding that the best inhibitory effect on both *Rel* was at 10 nM (either with RvE1 or LXA4, Figure S1A,B). This concentration was in accord with previous reports concerning stromal cells of the tendon and lung under inflammatory conditions.^40^, ^41^ The combination of RvE1 and LXA showed more significant suppression of *RelA* mRNA than either treatment alone (Figure 1B). The phosphorylation of p65 peaked at 24 h in LPS‐induced DPFs (Figure S1C), which was similar to some recent reports.^42^, ^43^ Therefore, we selected this incubating time in follow‐up experiments. The nuclear translocation of p65 is an important step of NF‐κB activation. In our present study, separate determination of p65 expression in the nucleus and cytoplasm was examined using western blotting. An increased ratio of nuclear to cytoplasmic p65 was observed in LPS‐induced DPFs, and some p65 protein translocated back to the cytoplasm after RvE1‐ or LXA4‐treatment, leading to a decrease in nuclear p65. The combination of RvE1 and LXA4 caused a further decline of nuclear p65 and a slight decrease in cytoplasmic p65 (Figure 2E); this simultaneous reduction of nuclear and cytoplasmic p65 is due to a significant decrease in *p65* transcription (Figure 1A). Meanwhile, the ratio of nuclear to cytoplasmic p65 was much lower than that with RvE1‐ or LXA4‐only treatment. Moreover, phosphorylation and acetylation of p65, which represent the duration and stable transcription of NF‐κB,^44^, ^45^ tended to be reduced more with the combination treatment (Figure 2F). As a result, combined administration exhibited greater suppression of pro‐inflammatory cytokines transcribed by NF‐κB than either RvE1 or LXA4 alone (Figure 1C–G). Our results were similar to other emerging studies of a combination of SPMs used in inflammation. For instance, Kantarci et al. found that a combination of RvE1 and LXA4 could more effectively resolve inflammation than RvE1‐ or LXA4‐only treatment in a murine model of Alzheimer's disease.^46^ In addition, Albuquerque‐Souza et al. employed a combination of MaR1 and RvE1 in LPS‐induced periodontal ligament stem cells, reversing the inflammatory condition and promoting regenerative properties.^47^

Further in vivo experiments verified the therapeutic effect of this combination seen in vitro. According to HE staining (Figure 5A) and CD11b immunofluorescence (Figure 5B) findings, single usage of RvE1 or LXA4 reduced infiltration of immune cells only partially. During repair in pulpitis or pulp injury, the ideal result is to form calcified bridges at the pulp exposure; notably, no ectopic mineralization was observed inside the dental pulp. Ectopic mineralization inside pulp has been demonstrated to be closely related to persistent infiltration of low‐grade inflammation in the pulp.^48^, ^49^ After RvE1‐ or LXA4‐only treatment, pulp inflammation was somewhat reduced but was still present at a low grade, causing ectopic mineralization inside the pulp as well. This finding is consistent with that of Abd‐Elmeguid et al.,^49^ who reported that ectopic calcification could be observed inside the inflamed dental pulp in reversible and irreversible pulpitis. In our present study, a large area of ectopic mineralization, which was positive for OCN (Figure 5C), was observed in pulpitis and RvE1‐ or LXA4‐only treatment groups on days 3 and 7, indicating that using RvE1 or LXA4 alone did not lead to complete resolution of pulpitis. Specifically, the combination of RvE1 and LXA4 promoted complete resolution, and the inflammatory response was limited to the pulp exposure area, leading to a lack of ectopic mineralization and the presence of only calcified tissue at the pulp exposure, thus representing the ideal result of vital pulp therapy (Figure 5). Immunohistochemistry staining of NLRP3, caspase‐1, IL‐1β and IL‐18 (Figure 6) also showed a similar tendency. These results suggested a superior pro‐resolving effect of the RvE1 and LXA4 combination. Previous reports have shown that RvE1 and LXA4 function through the ChemR23 and ALX receptors,^20^, ^50^, ^51^ respectively. In this study, we employed α‐NETA and BOC‐2 to inhibit ChemR23 and ALX to evaluate the impact on resolution. Inhibition of ChemR23 and ALX resulted in upregulated expression of *RelA* mRNA (Figure 3A) and phosphorylation and acetylation of p65 (Figure 3G), as well as expression of downstream pro‐inflammatory factors (Figure S2), indicating that RvE1 and LXA4 act synergistically in inhibition of NF‐κB, without mutual interference.

Furthermore, the molecular mechanism of the RvE1 and LXA4 combination on NF‐κB suppression was also investigated in this study. SIRTs are a highly conserved family of deacetylases, of which the enzymatic activities are strictly regulated by NAD+.^52^, ^53^ In LPS‐induced DPFs in vitro, we found that the expression of SIRT1 and SIRT6, which are located in the nucleus, was significantly reduced. These results are similar to those of well‐established studies in cardiovascular disease and vascular cells.^30^, ^31^ Particularly, our results also demonstrated expression of SIRT7 decreased in pulpitis and was consistent with a recent report regarding mastitis,^54^ in which activation of NF‐κB was increased as well, but in which the relationship of SIRT7 and NF‐κB remained unclear. Combined use of RvE1 and LXA4 could strongly increase the NAD+/NADH ratio (Figure 2A), resulting in marked elevation of the expression of the deacetylases SIRT1, SIRT6 and SIRT7 at the mRNA and protein levels, even to well above their levels in the control group (Figure 2B–D,F). Through inhibition of ChemR23 and/or ALX, the ratio of NAD+/NADH and the expression of SIRT1, SIRT6 and SIRT7 were reduced (Figure 3B–E), and the expression of p‐p65 and Ac‐p65 increased (Figure 3G). Furthermore, double‐immunofluorescence labelling was employed to evaluate the positional relationship of SIRT7 and NF‐κB. Representative images showed co‐localization of SIRT7 with high fluorescence intensity and p‐p65/Ac‐p65 with low fluorescence intensity in the nucleus after combined treatment with RvE1 and LXA4 (Figure 2G,H). Staining of samples from the in vivo experiment showed the same co‐localization result (Figure S3), indicating that high expression of SIRT7 may be closely related to NF‐κB inactivation. By silencing SIRT7 in DPFs, the pro‐resolving effect of RvE1 and LXA4 was largely abrogated, and the RelA mRNA level, ratio of nuclear to cytoplasmic p65 protein, and phosphorylation and acetylation of p65 were increased after combined RvE1 and LXA4 administration (Figure 4C,G–I). These results suggested that the upregulation of SIRT7 was an indispensable mechanism of the inhibitory effect of NF‐κB on RvE1 and LXA4 and may be a potential target for resolution of pulpitis. However, whether SIRT7 binds directly to NF‐κB, and the location of its binding site, remain to be studied. Interestingly, the NAD+/NADH ratio and the expression of SIRT1 and SIRT6 were observed to be decreased in combined‐treated si‐SIRT7 DPFs (Figure 4D–F), demonstrating that SIRT7 may play a synergistic role with SIRT1 and SIRT6 in NF‐κB inhibition and inflammation resolution.

The present study provides some insights regarding the mechanisms for inhibiting NF‐κB activation, which has been seen as a central pathway during pulpitis development. However, further preclinical and clinical trials are needed for verification of our findings. Moreover, future studies on the molecular mechanism of RvE1 and LXA4 combination treatment are necessary identify further potential targets for resolution of pulpitis and other infectious inflammatory conditions.

**FIGURE 7:**
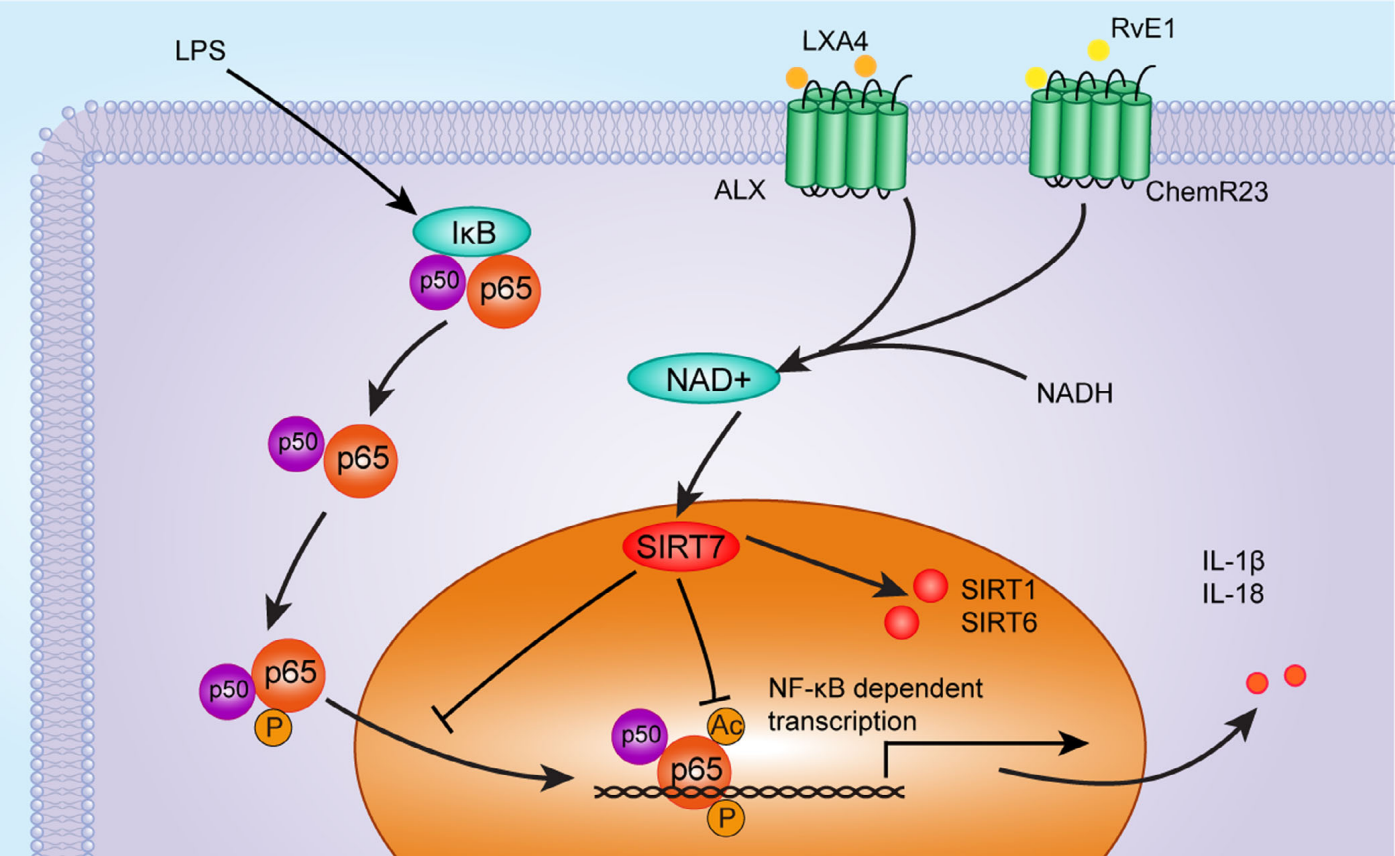
The molecular mechanism by which the RvE1 and LXA4 combination resolves pulpitis as proposed in the present study. LXA4, lipoxin A4; RvE1, resolvin E1

A visual summary of this study is presented in Figure 7. Based on the above findings, we propose that using a combination of SPMs may be a promising therapeutic strategy against pulpitis and that combined use of RvE1 and LXA4 shows great potential. Through in vitro and in vivo experiments, it was confirmed that the combination of RvE1 and LXA4 could effectively inhibit NF‐κB activation by dephosphorylation and deacetylation. At a molecular level, we clarified that SIRT7 was involved in resolution mediated by combination of RvE1 and LXA4 and may affect the expression of SIRT1 and SIRT6, indicating that they may synergistically promote inflammation resolution.

## CONFLICT OF INTEREST

The authors declare no conflict of interest.

## AUTHOR CONTRIBUTIONS

Qi Zhang conceived the project. Xiaochen Liu prepared all the materials, conducted the in vitro experiments, and analysed the samples of in vivo experiment. Chunmeng Wang established the animal model and performed the in vivo experiments. Liping Pang and Liangliang Pan performed the in vitro experiments. All authors discussed the results and commented. Xiaochen Liu completed the first draft and drew the schematic figures. All authors modified and Qi Zhang finalized the manuscript.

## Supporting information


**FIGURE S1** (A) Expression of *RelA* mRNA in LPS‐induced DPFs after treatment with RvE1 at concentrations of 1, 10, 100, and 1000 nM. (B) Expression of *RelA* mRNA in LPS‐induced DPFs after treatment with LXA4 at concentrations of 1, 10, 100, and 1000 nM. (C) Phosphorylation levels of p65 in DPFs were tested by western blotting after stimulation with 1 μg/ml of LPS for 3, 6, 12, 24, and 48 h.
**FIGURE S2** Effects on pro‐inflammatory factor expressions after ChemR23 and/or ALX inhibition. Inhibiting efficiency of (A) *ALX* and (B) *ChemR23* by BOC‐2 and α‐NETA. (C) *NLRP3*, (D) *Caspase1*, (E) *IL‐1β* and (F) *IL‐18* mRNA expressions detected by qPCR, and (G) their protein levels tested by western blot. (H) IL‐1β and (I) IL‐18 protein levels from the culture supernatant detected by ELISA. (*p < 0.05 and **p < 0.01)
ELISA, enzyme‐linked immunosorbent assay; qPCR, quantitative polymerase chain reaction
**FIGURE S3** Double‐immunofluorescence labelling images of day 3 samples. (A) Representative double‐immunofluorescence labelling images of SIRT7 (green) and Ac‐p65 (red), and the nuclei were stained with DAPI (blue). (B) Representative double‐immunofluorescence labelling images of SIRT7 (green) and p‐p65 (red), and the nuclei were stained with DAPI (blue).

## Data Availability

Some or all data that support the findings of this study are available from the corresponding author upon reasonable request.
