# Supplementary material for: Combination of resolvin E1 and lipoxin A4 promotes the resolution of pulpitis by inhibiting NF‐κB activation through upregulating sirtuin 7 in dental pulp fibroblasts
Source: Cell Prolif. 2022 Apr 11;55(5):e13227. doi: 10.1111/cpr.13227 (PMC9136498; doi:10.1111/cpr.13227)
Supplement: Supplementary file 1 — FIGURE S1 (A) Expression of RelA mRNA in LPS‐induced DPFs after treatment with RvE1 at concentrations of 1, 10, 100, and 1000 nM. (B) Expression of RelA mRNA in LPS‐induced DPFs after treatment with LXA4 at concentrations of 1, 10, 100, and 1000 nM. (C) Phosphorylation levels of p65 in DPFs were tested by western blotting after stimulation with 1 μg/ml of LPS for 3, 6, 12, 24, and 48 h. FIGURE S2 Effects on pro‐inflammatory factor expressions after ChemR23 and/or ALX inhibition. Inhibiting efficiency of (A) ALX and (B) ChemR23 by BOC‐2 and α‐NETA. (C) NLRP3, (D) Caspase1, (E) IL‐1β and (F) IL‐18 mRNA expressions detected by qPCR, and (G) their protein levels tested by western blot. (H) IL‐1β and (I) IL‐18 protein levels from the culture supernatant detected by ELISA. (*p < 0.05 and **p < 0.01) ELISA, enzyme‐linked immunosorbent assay; qPCR, quantitative polymerase chain reaction FIGURE S3 Double‐immunofluorescence labelling images of day 3 samples. (A) Representative double‐immunofluorescence labelling images of SIRT7 (green) and Ac‐p65 (red), and the nuclei were stained with DAPI (blue). (B) Representative double‐immunofluorescence labelling images of SIRT7 (green) and p‐p65 (red), and the nuclei were stained with DAPI (blue). [file CPR-55-e13227-s001.docx]

**Supplementary information:**

**Combination of resolvin E1 and lipoxin A4 promotes the resolution of pulpitis by inhibiting NF-κB activation through up-regulating sirtuin 7 in dental pulp fibroblasts**

Xiaochen Liu, Chunmeng Wang, Liping Pang, Liangliang Pan, Qi Zhang*

* Corresponding author

Department of Endodontics, School & Hospital of Stomatology, Tongji University, Shanghai Engineering Research Center of Tooth Restoration and Regeneration, 399 Middle Yanchang Road, Shanghai 200072, China

**Running title: RvE1 and LXA4 in pulpitis resolution**

**Keywords**: Dental pulp fibroblasts; Resolvin E1; Lipoxin A4; Pulpitis; Sirtuin 7

**Corresponding Author**:

Qi Zhang, PhD

Department of Endodontics, School & Hospital of Stomatology, Tongji University, Shanghai Engineering Research Center of Tooth Restoration and Regeneration, 399 Middle Yanchang Road, Shanghai 200072, China

Phone: (86-21) 66315500

E-mail: [qizhang@tongji.edu.cn](mailto:qizhang@tongji.edu.cn)

**
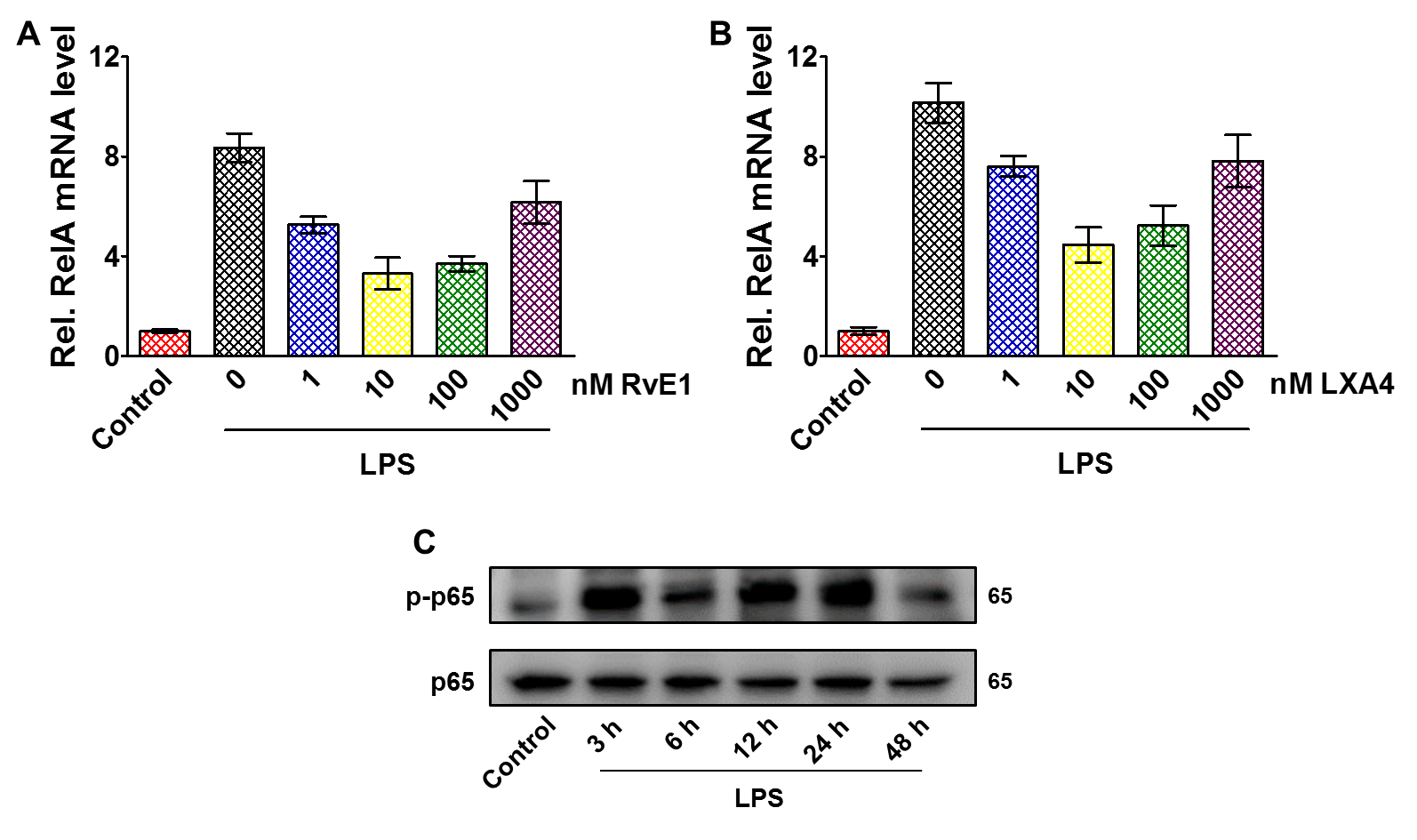
FIGURE S1** (A) Expression of *RelA* mRNA in LPS-induced DPFs after treatment with RvE1 at concentrations of 1, 10, 100, and 1000 nM. (B) Expression of *RelA* mRNA in LPS-induced DPFs after treatment with LXA4 at concentrations of 1, 10, 100, and 1000 nM. (C) Phosphorylation levels of p65 in DPFs were tested by western blotting after stimulation with 1 μg/ml of LPS for 3, 6, 12, 24, and 48 h.

**
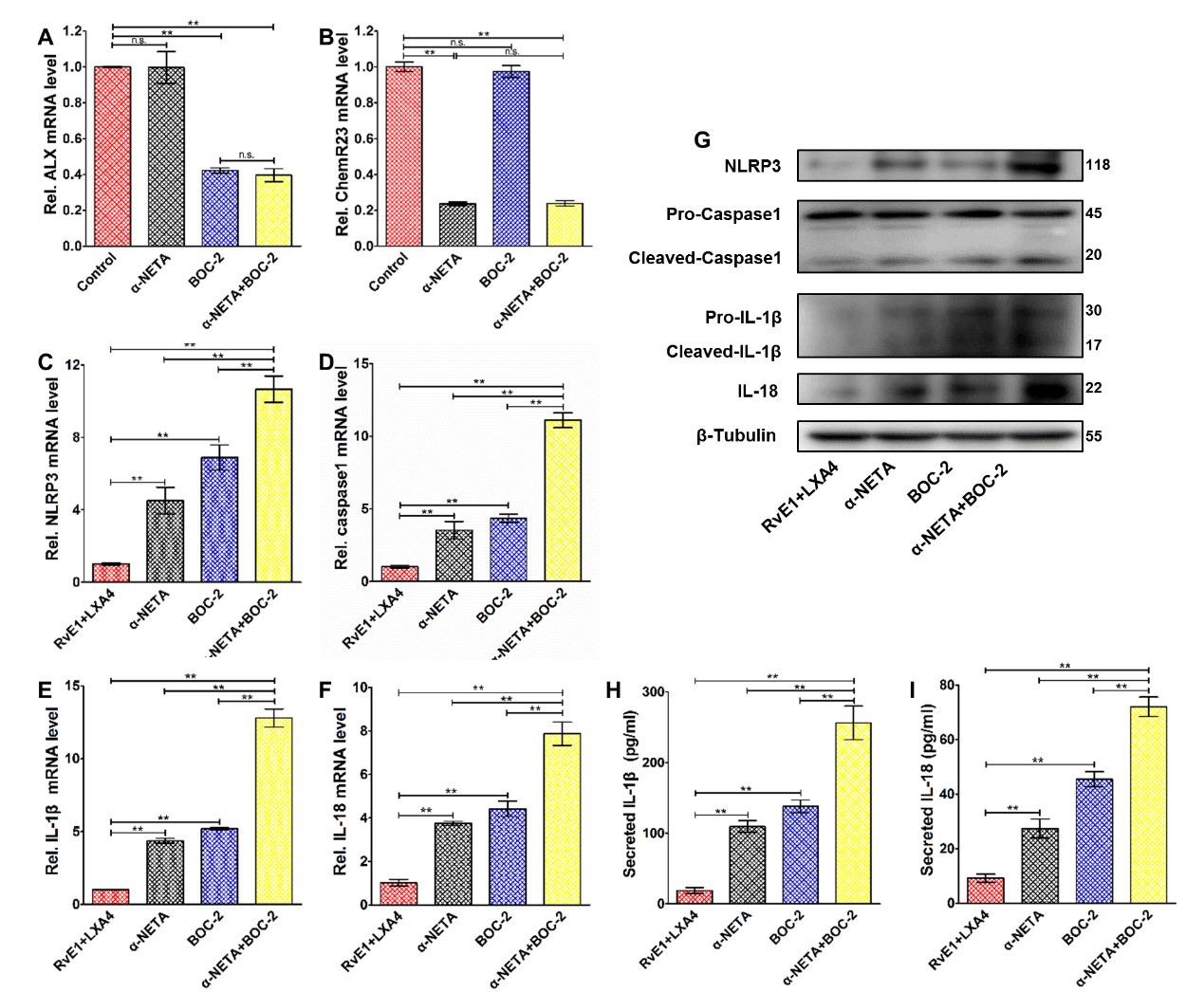
**

**FIGURE S2** Effects on pro-inflammatory factor expressions after ChemR23 and/or ALX inhibition. Inhibiting efficiency of (A) *ALX* and (B) *ChemR23* by BOC-2 and α-NETA. (C) *NLRP3*, (D) *Caspase1*, (E) *IL-1β* and (F) *IL-18* mRNA expressions detected by qPCR, and (G) their protein levels tested by western blot. (H) IL-1β and (I) IL-18 protein levels from the culture supernatant detected by ELISA. (**p*< 0.05 and ***p* < 0.01)

ELISA, enzyme-linked immunosorbent assay; qPCR, quantitative polymerase chain reaction


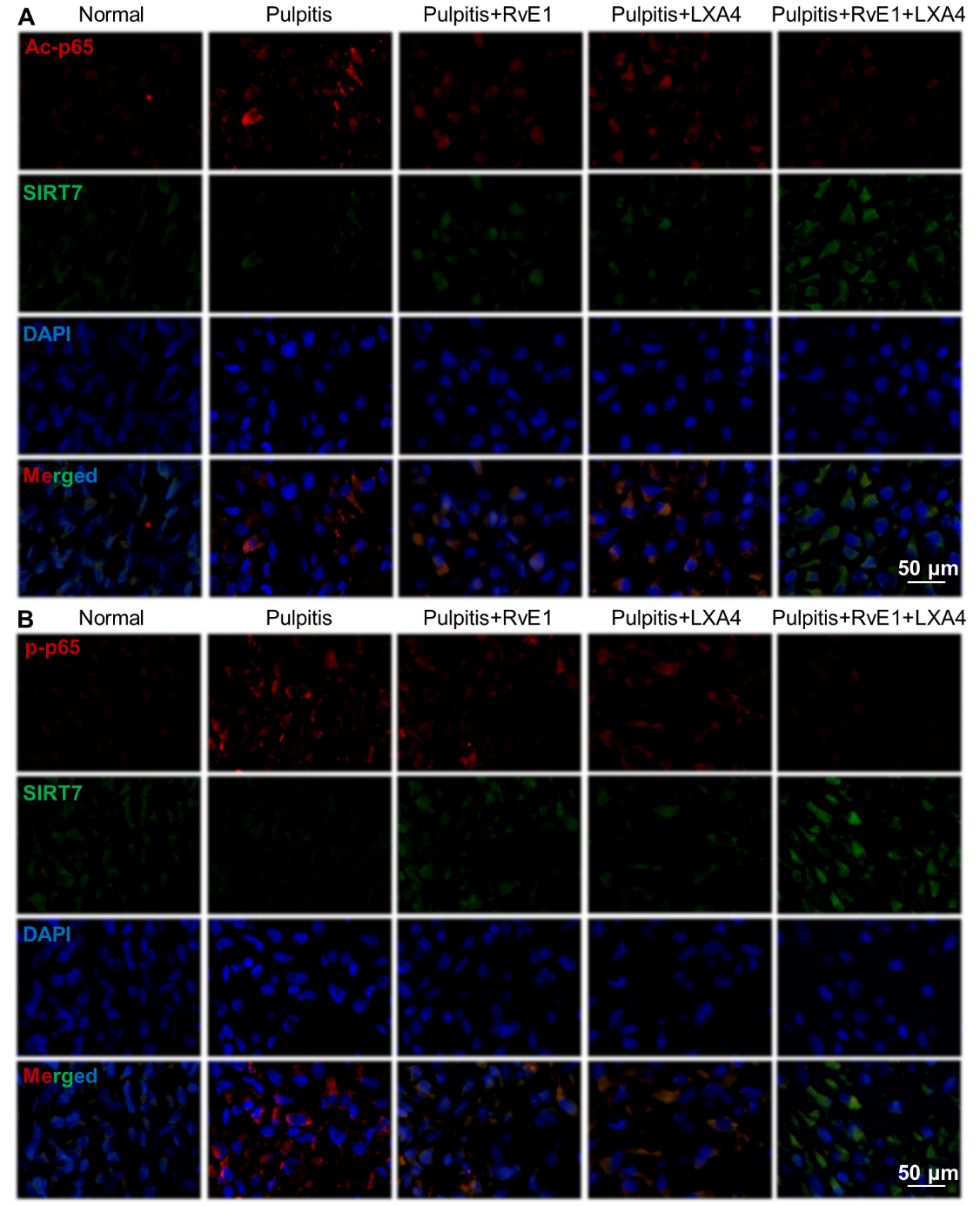


**FIGURE S3** Double-immunofluorescence labeling images of day 3 samples. (A) Representative double-immunofluorescence labeling images of SIRT7 (green) and Ac-p65 (red), and the nuclei were stained with DAPI (blue). (B) Representative double-immunofluorescence labeling images of SIRT7 (green) and p-p65 (red), and the nuclei were stained with DAPI (blue).
